# Polydatin Attenuates Intra-Uterine Growth Retardation-Induced Liver Injury and Mitochondrial Dysfunction in Weanling Piglets by Improving Energy Metabolism and Redox Balance

**DOI:** 10.3390/antiox11040666

**Published:** 2022-03-30

**Authors:** Yanan Chen, Yue Li, Peilu Jia, Shuli Ji, Hao Zhang, Tian Wang

**Affiliations:** 1 College of Animal Science & Technology, Nanjing Agricultural University, Nanjing 210095, China; 2019205019@njau.edu.cn (Y.C.); 2018105059@njau.edu.cn (P.J.); 2018105058@njau.edu.cn (S.J.); tianwang@njau.edu.cn (T.W.); 2 Institute of Animal Science, Jiangsu Academy of Agricultural Sciences, Nanjing 210014, China; 20180056@jaas.ac.cn

**Keywords:** energy metabolism, intra-uterine growth retardation, liver injury, mitochondrial dysfunction, weanling piglet, polydatin, redox balance

## Abstract

The present study investigated the potential of polydatin to protect against liver injury and the mitochondrial dysfunction of weanling piglets suffering from intra-uterine growth retardation (IUGR). Thirty-six normal birth weight weanling piglets and an equal number of IUGR littermates were given a basal diet with or without polydatin (250 mg/kg) from 21 to 35 d of age. Plasma and liver samples were collected to measure biochemistry parameters at 35 d of age. IUGR caused hepatic apoptosis, mitochondrial dysfunction, and oxidative damage, along with a lower efficiency of energy metabolism and inferior antioxidant ability. Polydatin decreased apoptotic rate, improved the features of mitochondrial damage, inhibited mitochondrial swelling and superoxide anion formation, and preserved mitochondrial membrane potential in the liver. Concurrently, polydatin promoted mitochondrial biogenesis, increased sirtuin 1 activity, and upregulated the expression levels of several genes related to mitochondrial function and fitness. Polydatin also facilitated mitochondrial oxidative metabolism with a beneficial outcome of increased energy production. Furthermore, polydatin mitigated the IUGR-induced reduction in manganese superoxide dismutase activity and prevented the excessive accumulation of oxidative damaging products in the liver. These findings indicate that polydatin confers protection against hepatic injury and mitochondrial dysfunction in the IUGR piglets by improving energy metabolism and redox balance.

## 1. Introduction

Intra-uterine growth retardation (IUGR) is a pathological complication with a reduced potential of fetal growth due to uteroplacental insufficiency, and it affects 5–10% of births with even greater rates in developing countries [1]. In swine production, this problem has been exacerbated over the last decades because of genetic selection toward sows with higher prolificacy as a means to increase profitability [2]. Compared with the normally growing piglets, IUGR piglets exhibit impaired development and metabolic function of the liver. In most cases, uteroplacental insufficiency curtails oxygen and nutrient transport from the mother to the fetus, resulting in a hemodynamic adaptation within the fetal body that benefits the cerebral circulation at the expense of reduced blood flow to the liver [3]. Nutritionally impaired growth, particularly of the liver, has long-term consequences on biosynthesis, metabolism, and host defense after birth [4]. Thus, IUGR piglets suffer from higher incidences of neonatal morbidity and mortality than their healthy littermates, economically decreasing the whole production efficiency [5,6].

Mitochondrial dysfunction has been identified as a major factor linked to the evolution of early liver disorders and later metabolic abnormalities in IUGR individuals [7,8]. Mitochondrial morphology and function are particularly susceptible to alterations in the surrounding cellular milieu, such as limited metabolic flux and various harmful stimuli, leading to disturbances in cellular bioenergetics and metabolic homeostasis [9]. Moreover, mitochondria are regarded as the primary producer of intracellular reactive oxygen species (ROS) [10]. Damage to mitochondrial function would exacerbate the leakage of electrons from the electron transport chain (ETC) and increase the generation of superoxide radicals, a precursor of most other ROS, including hydrogen peroxide and hydroxyl radicals [11]. Excessive ROS accumulation could induce oxidative damage to cellular macromolecules, such as lipids, proteins, and nucleic acids, leading to oxidative stress, a pathogenic state that is both a cause and a consequence of numerous liver diseases [12]. Therefore, appropriate interventions targeting mitochondrial dysfunction may have a beneficial role in improving the liver health of IUGR animals.

Recently, several attempts have been tried to protect the liver disorders of IUGR piglets by supplementation of different phytochemicals [13,14]. Curcumin, a polyphenol extracted from the herb *Curcuma longa*, has been found to mitigate lipid peroxidation and prevent ROS accumulation in the liver of IUGR piglets [13]. Dihydroartemisinin, a semisynthetic analog of artemisinin with favorable antioxidant activity, could alleviate IUGR-induced hepatic oxidative damage by promoting the activity of total superoxide dismutase [14]. However, the effect of these bioactive substances on mitochondrial function is less explored, and more accurate interventions with the potential to attenuate mitochondrial dysfunction in the liver of IUGR piglets need to be identified.

Polydatin is a naturally occurring compound belonging to stilbenes, a class of molecules that share a common 1,2-diphenylethylene nucleus. Structurally, polydatin is a glucoside of resveratrol where the glucoside group bonded in position C-3 substitutes a hydroxyl group [15]. In nature, polydatin is the most abundant form of resveratrol [16], and its average concentration in the Polygonum cuspidatum and red wine is approximately ten times greater than resveratrol [17]. Polydatin shares a host of pharmacological similarities with resveratrol, including antioxidation and anti-inflammation [18]. Notably, different from resveratrol, which penetrates the cell passively, polydatin can enter the cell via an active mechanism using glucose carriers [19]. In addition, polydatin has greater resistance to enzymatic oxidation than resveratrol in vivo [20]. These properties endow polydatin with a higher absorption rate and better bioavailability with respect to resveratrol [21]. Thus, polydatin has recently attracted much attention in animal research [22,23,24].

Emerging studies have suggested that polydatin could serve as a novel mitochondrial protector to mitigate the features of mitochondrial dysfunction [23,25,26]. Zeng et al. [25] have shown that polydatin preserves mitochondrial morphology and promotes energy supply in a model of intestinal hemorrhagic shock. In the studies of diabetic neuropathy, polydatin also facilitates mitochondrial biogenesis and oxidative metabolism possibly by stimulating sirtuin 1 (SIRT1) and nuclear factor erythroid 2-related factor 2 (NRF2) axis [26]. However, no sufficient information on the potential of polydatin to alleviate IUGR-induced hepatic mitochondrial dysfunction is available. Therefore, the aim of this investigation was to evaluate the effects of dietary supplementation with polydatin on the hepatic damage, mitochondrial function, energy metabolism, and redox status of weanling piglets suffered from IUGR.

## 2. Materials and Methods

### 2.1. Ethical Approval

The present study was carried out with approval from the Nanjing Agricultural University Institutional Animal Care and Use Committee (Permit number SYXK-2017-0027).

### 2.2. Experimental Design

At delivery, 36 litters, born to 3rd parity sows, in litters of 11 to 15 total born piglets were carefully selected. On the basis of birth weights, one IUGR female piglets (0.91 ± 0.10 kg) and one same-sex normal birth weight (NBW; 1.56 ± 0.08 kg) littermates were obtained from each of the 36 litters. The criteria used at selection were based on the methods as reported previously [7,27]. IUGR piglets were identified by a birth weight below 2 standard deviations of the arithmetic mean of the herd, while those with a birth weight near the mean value (less than 0.5 standard deviation) were defined as NBW piglets. All piglets were kept in a farrowing room and fed by sows freely during the suckling period (1 to 21 d of age). After weaning, the piglets were moved to the weaner unit. Half of the NBW (N) and IUGR (I) piglets were given a basal diet (C) for 14 d, while the residual piglets were fed a diet supplemented with 250 mg polydatin (P) per kg of diet. The polydatin was purchased from Aladdin Reagents Co., Ltd. (Shanghai, China), and its purity was 97.36%, determined by high-performance liquid chromatography (HPLC; Figure S1). Thus, all piglets were allocated to four treatment groups (i.e., NC, IC, NP, or IP) in a 2 × 2 factorial design. Each treatment group (18 piglets) consisted of six replicates, with three piglet per replicate (*n* = 6). The basal diet was formulated based on the recommendations of the National Research Council (2012) for piglets weighing 5–10 kg (Table S1). All piglets had free access to food and water during the feeding trial.

### 2.3. Sample Collection

At 35 d of age, one piglet was randomly chosen from each replicate. Blood samples were collected from the anterior vena cava of piglets. Following which, piglets were killed by exsanguination after electrical stunning and the liver samples were collected immediately. Liver tissues from the left lobe were fixed in 2.5% glutaraldehyde or 4% paraformaldehyde in phosphate buffer saline (PBS). A portion of the fresh liver was used to extract mitochondrial and nuclear proteins. The remainder of liver samples was snap-frozen in liquid nitrogen and stored at −80 °C for subsequent biochemical analyses.

### 2.4. Determination of Plasma Biochemical Parameters

Plasma samples were separated by centrifugation after sampling immediately. Thereafter, plasma glucose (GLU), triglycerides (TG), total cholesterol (TC), total protein (TP), urea nitrogen (UN), total bilirubin (T-Bil), alanine aminotransferase (ALT), and aspartate aminotransferase (AST) levels were determined with an automatic biochemical analyzer (Selectra XL; Vital scientific, Huizen, The Netherland). All the assay kits were purchased from the Nanjing Jiancheng Bioengineering Institute (Nanjing, China).

### 2.5. Hepatic Apoptosis

The number of apoptotic cells in the liver of piglets was assessed by the terminal deoxynucleotidyl transferase-mediated dUTP nick-end labeling (TUNEL) method using a BrightGreen Apoptosis Detection Kit (Vazyme, Nanjing, China). The paraffin-embedded liver slices were deparaffinized by ethanol solutions. Then, the slices were partially digested with proteinase K solution (20 μg/mL; Servicebio Technology, Wuhan, China). Sections were washed with PBS and covered with TUNEL-mixed reagents in a light-protected container at 37 °C. After 1 h of incubation, the liver slices were counterstained slightly with 4′-6-diamidino-2-phenylindole (DAPI) to label the nuclei and then imaged by a fluorescence microscope (Nikon Eclipse C1; Nikon, Tokyo, Japan). The TUNEL-positive cells were quantified in fifteen random fields per section.

### 2.6. Hepatic Caspase Activities

Caspase-3, -8, and -9 activities of liver samples were detected with colorimetric assay kits (Beyotime Institute of Biotechnology, Haimen, China). The frozen liver tissues were lysed, and the lysates were centrifuged at 16,000× *g* at 4 °C for 15 min to collect the supernatant. Then, the supernatant samples were incubated with detection buffer and corresponding caspase-specific substrates at 37 °C for 2 h. After that, the hydrolysis products were monitored at 405 nm using a Multiskan SkyHigh (Thermo Fisher Scientific, Waltham, MA, USA) to calculate caspase activities.

### 2.7. Electron Microscopy

Liver pieces were fixed in 2.5% glutaraldehyde solution for 24 h and then post-fixed in 1% osmic acid. Thereafter, they were dehydrated in a graded series of alcohol and finally embedded in pure Epon. Samples were cut into super-thin sections, placed on copper grids, and doubly stained with urinal acetate and lead citrate. After that, the sections were photographed with a transmission electron microscopy (TEM; Hitachi H-7650, Hitachi, Tokyo, Japan).

### 2.8. Mitochondrial Separation

Mitochondrial proteins were purified from fresh liver tissues using a Tissue Mitochondria Isolation Kit from Beyotime Institute of Biotechnology (Haimen, China). Approximately 200 mg of liver tissues were homogenized in a separation buffer. The homogenate was centrifuged at 600× *g* for 15 min and the resulting supernatant was recentrifuged at 12,000× *g* for 15 min. The pellets were washed using sucrose solution and then centrifuged for 15 min at 12,000× *g* to obtain mitochondrial enriched pellets. Protein concentrations of the collected mitochondrial samples were quantified using a Bicinchoninic Acid Protein Assay Kit (Beyotime Institute of Biotechnology, Haimen, China).

### 2.9. Mitochondrial Swelling

The calcium-triggered mitochondrial swelling assay was performed following the previous methods [28]. Mitochondrial pellets isolated from fresh liver tissues were suspended in 1 mL of working buffer (pH 7.2) containing 150 mM KCl, 10 mM Tris, 2 mM K_2_HPO_4_, 5 mM glutamate, and 5 mM malate. Swelling was induced by the addition of 200 μM CaCl_2_. The levels of mitochondrial swelling were assessed by continuously measuring changes in absorbance at 540 nm on the microplate spectrophotometer (Multiskan SkyHigh, Thermo Fisher Scientific, Waltham, MA, USA) at 37 °C for 10 min. Measures were taken every 60 s with 20 s strong shaking in the interval to avoid mitochondria deposition at the bottom of the well.

### 2.10. Mitochondrial Superoxide Anion

Dihydroethidium (DHE; Thermo Fisher Scientific, Waltham, MA, USA) is an oxidant-sensitive fluorescent probe that can be used for detection of mitochondrial superoxide anion [29]. DHE is oxidized by superoxide anion to yield a red fluorescent product, which can be detected at Ex./Em. = 300/610 nm. Briefly, approximately 100 μg of mitochondrial protein were suspended in 2 mL of assay buffer (pH 7.4) containing 5 mM Hepes, 210 mM mannitol, and 70 mM sucrose, supplemented with 10 mM succinate or 2 μM rotenone. Then, the working solution was stained with 10 μM DHE for 30 min at 37 °C in the dark. Changes in fluorescence were determined using a fluorospectrophotometer (Hitachi F-7000, Hitachi, Tokyo, Japan).

### 2.11. Mitochondrial DNA (mtDNA) Content

Genomic DNA was isolated from liver samples using a FastPure Cell/Tissue DNA Isolation Mini Kit (Vazyme, Nanjing, China). The copy number of mtDNA was detected by co-amplifying the mt D-loop, using the nuclear-encoded beta actin (ACTB) as a housekeeping gene. Primers for mt D-loop and ACTB genes are listed in Table 1. Real-time PCR was performed using the ChamQ^TM^ SYBR^®^ qPCR Master Mix Kit (Vazyme, Nanjing, China) on a QuantStudio 5 Real-time PCR System (Applied Biosystems, Foster City, CA, USA). The values of relative quantification were calculated using the 2^−ΔΔCt^ method [30].

### 2.12. Immunofluorescence Staining of Hepatic SIRT1 Protein

Protein expression of SIRT1 in the liver was determined by immunofluorescent staining. Briefly, liver paraffin-embedded sections with 5-μm thickness were deparaffinized and rehydrated. The slides were incubated with an Antigen Retrieval Solution (Servicebio Technology, Wuhan, China) at 100 °C for 15 min. After inhibition of endogenous peroxidase by PBS containing 1% hydrogen peroxide for 10 min, the liver sections were incubated with 5% bovine serum albumin in PBS for 30 min to minimize nonspecific staining. Sections were then incubated with the primary antibody against SIRT1 (1:100; Proteintech, Chicago, IL, USA) in a humidified chamber overnight at 4 °C. After washing, the liver sections were treated with a FITC-conjugated secondary antibody in a dark location at 25 °C for 1 h, following which nuclei were stained with DAPI in anti-fade mounting medium (Servicebio Technology, Wuhan, China). The images of all liver sections were captured using a fluorescence microscope (Nikon Eclipse C1). Ten random fields per specimen were quantified using Image J software (NIH, Baltimore, MD, USA).

### 2.13. Hepatic SIRT1 Activity

Nuclear extracts were prepared from fresh liver tissues with a Nuclear Protein Extraction Kit (Solarbio, Beijing, China). The protein concentration of nuclear extracts was quantified. A specific deacetylase detection kit was then used to determine SIRT1 activity of the nuclear extracts, as the procedures of the manufacturer (Genmed Scientifics Inc., Shanghai, China).

### 2.14. Mitochondrial Oxidative Enzyme Activities

Mitochondrial proteins were purified from fresh liver tissues using a Tissue Mitochondria Isolation Kit from Beyotime Institute of Biotechnology (Haimen, China). Citrate synthase (CS), isocitrate dehydrogenase (ICDH), alpha ketoglutarate dehydrogenase (α-KGDH), and malic dehydrogenase (MDH) activities in the liver mitochondria were assessed with commercial kits purchased from Suzhou Comin Biotechnology Co., Ltd. (Suzhou, China). Detection kits for complexes I–IV and adenosine triphosphate (ATP) synthase activities were obtained from Solarbio. All procedures were performed strictly according to the manufacturers’ instructions. The results were normalized to the total protein content in each sample.

### 2.15. Hepatic ATP Content

The frozen liver samples were cut into pieces, crushed, and blended with the ATP lysis buffer to a final concentration of 10%. The lysates were centrifuged at 12,000× *g* for 5 min at 4 °C. The supernatants were used to determine the levels of ATP using an ATP Bioluminescence Assay Kit (Beyotime Institute of Biotechnology, Haimen, China). An aliquot of working solution (200 μL) was added to 40 μL of liver lysates, and the luminescence was immediately determined on a Promega GloMax 2020 Single Tube Luminometer (Promega, Madison, WI, USA). The amount of ATP was calculated based on ATP standards and normalized to the concentration of total protein in each sample.

### 2.16. Hepatic Nicotinamide Adenine Dinucleotide (NAD^+^) and Its Reduced Form (NADH)

Hepatic NAD^+^ and NADH extraction and subsequent quantitative analysis were performed according to the methods of Goldman et al. [31]. Frozen liver samples (typically 180–200 mg) were homogenized in 2.0 mL of 0.6 M HClO_4_ on ice and precipitated by the addition of 1 M NaOH with shaking for 2 min. The mixture was centrifuged at 12,000× *g* for 5 min at 4 °C and filtered using 0.45-μm membrane filters (Millipore, Bedford, MA, USA). After that, metabolites were analyzed using a HPLC system (UltiMate™ 3000; Thermo Fisher Scientific, Waltham, MA, USA) equipped with a UV detector and a reverse-phase column (Agilent ZORBAX Eclipse Plus C18; 5 μm, 250 × 4.6 mm; Agilent Technologies, Palo Alto, CA, USA). Chromatography conditions were set as follows: UV detection, 254 nm; injection volume, 10 μL; column temperature, 40 °C; mobile phase, consisting of 215 mM KH_2_PO_4_, 1.2 mM tetrabutylammonium bisulfate, and 10% methanol; flow-rate, 1.0 mL/min. Peaks were identified by their retention time using authentic standards, and the contents of NAD^+^ and NADH in the liver samples were normalized to the wet weight of each liver sample.

### 2.17. Evaluation of In Vitro Antioxidant Activity of Polydatin

The capacities of several antioxidants, including polydatin, to scavenge 2,2-dipheny-l-picrylhydrazyl (DPPH) free radicals were measured according to the method of Liu et al. [32]. Additional details can be found in the Supplementary Materials.

The alpha mouse liver 12 (AML-12) cell line, an in vitro model system, was used to further evaluated the effects of polydatin on cell viability and copper/zinc superoxide dismutase (Cu/Zn-SOD) and manganese superoxide dismutase (Mn-SOD) activities under the conditions of oxidative stress. A Cell Counting Kit-8 Assay Kit (Yeasen; Shanghai, China) was used to determine cell viability. The activity detection kits for Cu/Zn-SOD and Mn-SOD were purchased from the Nanjing Jiancheng Bioengineering Institute (Nanjing, China). Further details concerning cell culture, treatment, and experimental procedures can be found in the Supplementary Materials.

### 2.18. Hepatic Antioxidant Enzyme Activities and Redox Metabolites

Frozen liver tissues were homogenized in 1:9 (*w*/*v*) ice-cold saline (0.86%) and then centrifuged at 4500× *g* for 20 min at 4 °C. The effects of polydatin on the hepatic antioxidant capacity of weaned piglets with different birth weights were evaluated by detecting the content of reduced glutathione (GSH) and the activities of Cu/Zn-SOD, Mn-SOD, glutathione peroxidase (GPx), glutathione reductase (GR), and catalase (CAT). All corresponding detection kits were supplied by the Nanjing Jiancheng Bioengineering Institute (Nanjing, China). To evaluate the severity of hepatic oxidative damage, malondialdehyde (MDA) and protein carbonyl (PC) contents were detected with colorimetric kits, as the recommended procedures of respective instructions (Nanjing Jiancheng Bioengineering Institute, Nanjing, China). For detection of hepatic 8-hydroxy-2-deoxyguanosine (8-OHdG) levels, approximately 100 mg of liver tissues were used for the extraction of DNA with a FastPure Cell/Tissue DNA Isolation Mini Kit (Vazyme, Nanjing, China). The concentration of 8-OHdG in the isolated DNA samples was determined by enzyme-linked immunosorbent assay using a commercial kit (Cayman Chemical, Ann Arbor, MI, USA).

### 2.19. Total RNA Isolation and RT-qPCR Analysis

Total RNA in frozen liver samples was isolated using TRIzol reagent according to the recommended protocol of the manufacturer (Vazyme, Nanjing, China). After determining the concentration, purity, and integrity of RNA preparations, 1 μg of RNA was converted to complementary DNA using the HiScript III 1st Strand cDNA Synthesis Kit with gDNA Eraser (Vazyme, Nanjing, China). Real-time PCR was performed on a QuantStudio 5 Real-time PCR System (Applied Biosystems, Foster City, CA, USA). Details of the primer sequences for SIRT1, sirtuin 3 (SIRT3), peroxisome proliferator activated receptor gamma coactivator 1 alpha (PGC1α), nuclear respiratory factor 1 (NRF1), NRF2, estrogen related receptor alpha (ERRα), mitochondrial transcription factor A (TFAM), peroxisome proliferator activated receptor alpha (PPARα), carnitine palmitoyltransferase 1 alpha (CPT1α), polymerase gamma (POLG), fatty acid binding protein 1 (FABP1), acetyl-CoA acyltransferase 1 (ACAA1), acyl-CoA oxidase 1 (ACOX1), acyl-CoA synthetase long chain family member 1 (ACSL1), acyl-CoA synthetase long-chain family member 5 (ACSL5), hydroxyacyl-CoA dehydrogenase trifunctional multienzyme complex subunit alpha (HADHA), single-strand DNA-binding protein (SSBP1), Mn-SOD, peroxiredoxin 3 (PRDX3), peroxiredoxin 5 (PRDX5), thioredoxin reductase 2 (TXNRD2), ACTB, and glyceraldehyde-3-phosphate dehydrogenase (GAPDH) are listed in Table 1. The mRNA expression of the target genes was normalized to ACTB gene expression and calculated according to the 2^−ΔΔCt^ method [30].

### 2.20. Statistical Analysis

Statistical significances of the main factors (birth weight [BW] and diet) and their interaction were measured by two-way analysis of variance with general linear model procedure (SPSS Statistics; Version 26.0, IBM, Armonk, NY, USA). A *p*-value less than 0.05 was considered statistically significant. When the *p*-value of interaction between the main factors was less than 0.05, Tukey’s post hoc tests for multiple comparisons were performed to determine statistical significance among the groups. Results were expressed as mean values and standard deviations.

## 3. Results

### 3.1. Effects of BW and Diet Factors on Plasma Biochemical Parameters of Weaned Piglets

Compared with the NBW piglets, IUGR increased UN content (*p* = 0.041; Table 2) and ALT (*p* = 0.014) and AST activities (*p* = 0.015) in the plasma of piglets. Irrespective of the BW factor, dietary supplementation with polydatin enhanced plasma GLU (*p* = 0.041) and TG concentrations (*p* = 0.006) and inhibited plasma AST activity (*p* = 0.001) and T-Bil content (*p* = 0.039). No difference was observed in plasma TC or TP content among the groups (*p* > 0.05).

### 3.2. Effects of BW and Diet Factors on Hepatic Apoptosis Rate and Caspase Activities of Weaned Piglets

**Figure 1:**
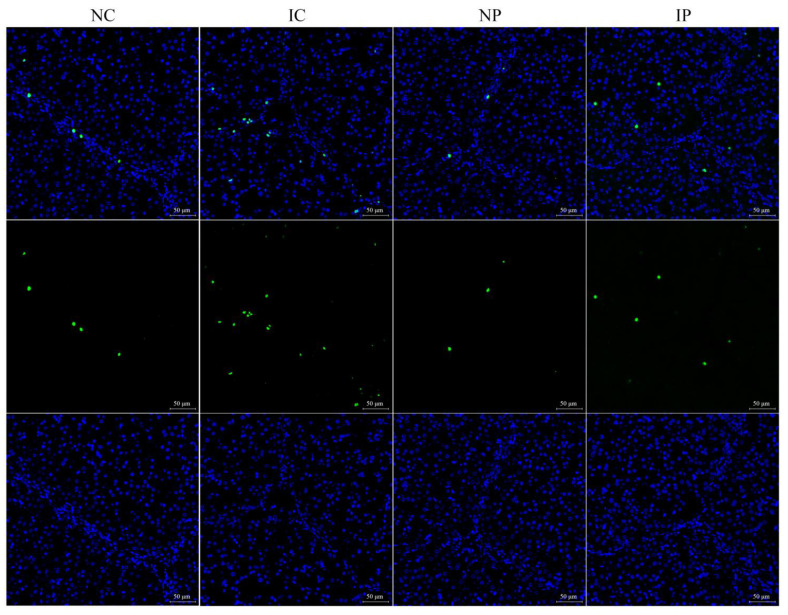
Representative photographs of TUNEL staining from liver sections. IC, intra-uterine growth retarded piglets fed a basal diet; IP, intra-uterine growth retarded piglets fed a polydatin-supplemented diet; NC, normal birth weight piglets fed a basal diet; NP, normal birth weight piglets fed a polydatin-supplemented diet; TUNEL, terminal deoxynucleotidyl transferase-mediated dUTP nick-end labeling.

The IUGR piglets showed increases in the number of apoptotic cells (*p* < 0.001, Figure 1 and Table 3) and the activities of caspase-3 (*p* = 0.016) and caspase-9 (*p* = 0.013) in the liver when compared with the NBW littermates. The apoptotic rate (*p* = 0.006) and caspase-9 activity (*p* = 0.004) were decreased in the liver of piglets fed a polydatin-supplemented diet in comparison with those fed a basal diet. Two-way ANOVA exhibited an interaction between BW and diet factors for the rate of apoptosis (*p* = 0.014) in the liver, and the excessive number of apoptotic cells induced by IUGR was restored to the normal levels after polydatin supplementation.

### 3.3. Effects of BW and Diet Factors on Hepatic Energy Metabolism of Weaned Piglets

Relative to the NBW piglets, the IUGR piglets exhibited decreased activities of mitochondrial CS (*p* = 0.014; Table 4), α-KGDH (*p* = 0.035), and ETC complexes I (*p* = 0.023) and III (*p* = 0.039) in the liver, in parallel with lower ATP content (*p* = 0.012) and NAD^+^/NADH ratio (*p* = 0.045). Contrarily, polydatin treatment facilitated the activities of mitochondrial CS (*p* = 0.026), α-KGDH (*p* = 0.046), complex I (*p* = 0.017), and ATP synthase (*p* = 0.027) in the liver of piglets, when compared with their counterparts fed a basal diet. The contents of ATP (*p* = 0.045) and NAD^+^ (*p* = 0.005), as well as the ratio of NAD^+^ to NADH (*p* = 0.047), were substantially elevated by polydatin supplementation. In addition, administration of polydatin ameliorated the IUGR-induced decreases in mitochondrial ICDH (*p* = 0.013) and ETC complex IV activities (*p* = 0.008). Furthermore, neither BW nor diet factor induced any obvious effects on mitochondrial MDH or complex II activity or NADH content in the liver of weaned piglets (*p* > 0.05).

### 3.4. Effects of BW and Diet Factors on Hepatic Ultrastructure of Weaned Piglets

**Figure 2:**
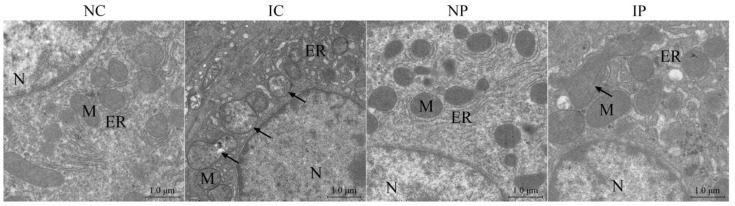
Effects of dietary polydatin supplementation on hepatic ultrastructure of normal birth weight and intra-uterine growth retarded weanling piglets. ER, endoplasmic reticulum; IC, intra-uterine growth retarded piglets fed a basal diet; IP, intra-uterine growth retarded piglets fed a polydatin-supplemented diet; N, nucleus; NC, normal birth weight piglets fed a basal diet; NP, normal birth weight piglets fed a polydatin-supplemented diet; M, mitochondrion.

The TEM revealed that hepatocytes from the NC and NP groups contained round nuclei, regular and intact mitochondria, and tightly packed endoplasmic reticulum (ER, Figure 2). However, in the liver of the IC group, quite a few hepatocytes showed ultrastructure injury, as characterized by the presence of swollen mitochondria with irregular shapes and disorganized cristae, which were rarely observed in the NC and NP groups. Additionally, the cisterns of hepatocellular ER were robustly dilated in the IC group compared with normally growing piglets. Although polydatin failed to mitigate ER swelling, most of the mitochondria in the liver of the IP group presented orderly cristae and reduced dilatation and vacuoles, indicating that polydatin may have the potential to rescue IUGR-induced mitochondrial damage.

### 3.5. Effects of BW and Diet Factors on Hepatic Mitochondrial Injury of Weaned Piglets

**Figure 3:**
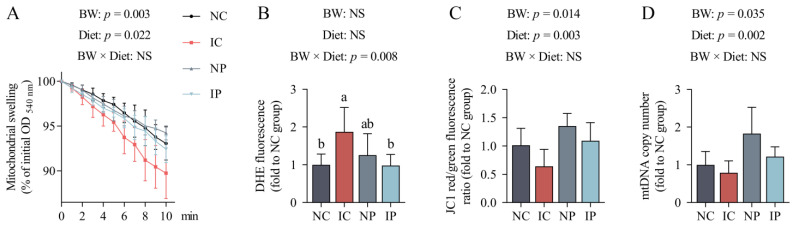
Effects of dietary polydatin supplementation on hepatic mitochondrial swelling (**A**), superoxide anion generation (**B**), membrane potential (**C**), and mtDNA copy number (**D**) of normal birth weight and intra-uterine growth retarded weanling piglets. DHE, dihydroethidium; IC, intra-uterine growth retarded piglets fed a basal diet; IP, intra-uterine growth retarded piglets fed a polydatin-supplemented diet; NC, normal birth weight piglets fed a basal diet; NP, normal birth weight piglets fed a polydatin-supplemented diet; NS, non-significant; mtDNA, mitochondrial DNA. ^a,b^ Data with different letters were significantly different (*p* < 0.05). Results are expressed as mean values and standard deviations represented by a vertical bar (*n* = 6).

The opening of the mitochondrial permeability transition pore (mPTP) was determined by Ca^2+^-induced mitochondrial swelling. As shown in Figure 3A, IUGR increased mitochondrial swelling (*p* = 0.003) in the liver of piglets. In contrast, polydatin treatment inhibited the swelling of mitochondria (*p* = 0.022) compared with the basal diet. Superoxide anion generation was increased in the hepatic mitochondria from the IC group compared with the NC group (*p* < 0.05; Figure 3B), but this effect was not observed in the presence of polydatin treatment (*p* > 0.05); these findings were reflected by the interaction between BW and Diet (*p* = 0.008).

Mitochondria from the IUGR livers exhibited a reduction in the fluorescence ratio of aggregates (red) to monomers (green) compared with the NBW piglets (*p* = 0.014; Figure 3C). The copy number of hepatic mtDNA (*p* = 0.035; Figure 3D) was also lower in the IUGR piglets in comparison with their NBW counterparts. Dietary supplementation with polydatin increased mitochondrial membrane potential (*p* = 0.003) and mtDNA content (*p* = 0.002) in the liver of piglets in comparison with their littermates fed a basal diet.

### 3.6. Effects of BW and Diet Factors on Hepatic SIRT1 Expression and Activity of Weaned Piglets

**Figure 4:**
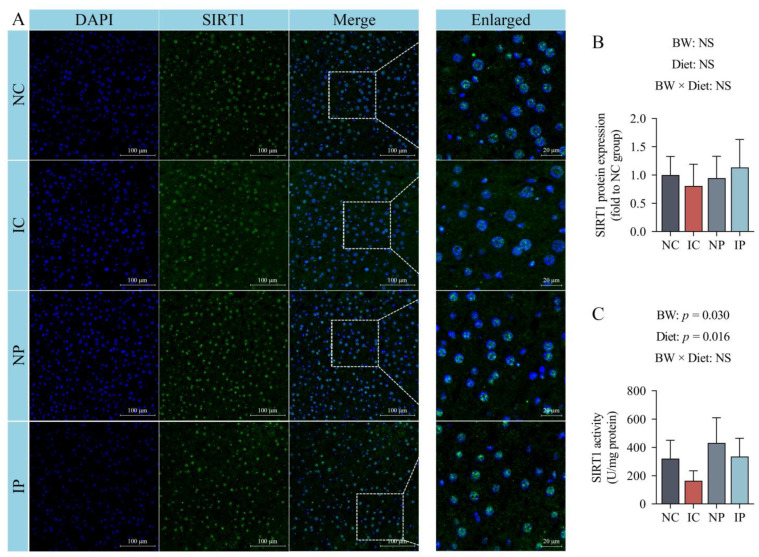
Effects of dietary polydatin supplementation on the protein expression (**A**,**B**) and activity (**C**) of sirtuin 1 in the liver of normal birth weight and intra-uterine growth retarded weanling piglets. DAPI, 4′-6-diamidino-2-phenylindole; IC, intra-uterine growth retarded piglets fed a basal diet; IP, intra-uterine growth retarded piglets fed a polydatin-supplemented diet; NC, normal birth weight piglets fed a basal diet; NP, normal birth weight piglets fed a polydatin-supplemented diet; NS, non-significant; SIRT1, sirtuin 1. Results are expressed as mean values and standard deviations represented by a vertical bar (*n* = 6).

The immunofluorescent staining failed to record any significant variation among the groups (*p* > 0.05; Figure 4A,B). In addition, a decrease in SIRT1 activity (*p* = 0.030; Figure 4C) was observed in the liver of IUGR piglets relative to their NBW littermates. Conversely, the piglets fed a polydatin-supplemented diet exhibited an increase in hepatic SIRT1 activity (*p* = 0.016) compared to that in their counterparts fed a basal diet.

### 3.7. Effects of BW and Diet Factors on Hepatic Redox Status of Weaned Piglets

We first conducted several in vitro experiments to evaluate the antioxidant activity of polydatin. Free radical scavenging assay showed that polydatin could act as a scavenger of DPPH and superoxide radicals (Figures S2 and S3). In cellular experiments, polydatin was found to increase the viability (*p* < 0.05; Figure S4) of AML-12 cells upon the exposure of hydrogen peroxide, and it also tended to increase the activity of cellular Mn-SOD (*p* = 0.090; Table S2). These findings support that polydatin could be a promising antioxidant.

The animal experiment indicated that the IUGR piglets had decreased Cu/Zn-SOD activity (*p* = 0.018; Table 5) and GSH content (*p* = 0.030), whereas they had increased levels of 8-OHdG (*p* = 0.005) and MDA (*p* = 0.046) compared to those in their NBW littermates. Compared with the basal diet, treatment with polydatin elevated Mn-SOD activity (*p* = 0.029) and inhibited 8-OHdG concentration (*p* = 0.011) in the liver. The decreased activity of Mn-SOD caused by IUGR was restored by polydatin supplementation (*p* = 0.041). Additionally, polydatin alleviated IUGR-induced increases in hepatic 8-OHdG (*p* = 0.004) and MDA contents (*p* = 0.024). However, neither BW nor diet factor had any effect on GPx, GR, or CAT activity (*p* > 0.05).

### 3.8. Effects of BW and Diet Factors on Hepatic Gene Expression of Weaned Piglets

The expression levels of hepatic PGC1α (*p* = 0.025, Table 6), TFAM (*p* = 0.005), FABP1 (*p* = 0.028), HADHA (*p* = 0.031), and PRDX5 (*p* = 0.028) were decreased in the IUGR piglets compared with their NBW littermates. Piglets fed a diet supplemented with polydatin had upregulated levels of SIRT3 (*p* = 0.048), PGC1α (*p* = 0.003), NRF1 (*p* = 0.025), ERRα (*p* = 0.010), TFAM (*p* = 0.043), PPARα (*p* < 0.001), CPT1α (*p* = 0.001), FABP1 (*p* < 0.001), ACSL5 (*p* = 0.001), and HADHA mRNA (*p* = 0.023) compared with those fed a basal diet. A similar effect was also noted for the mRNA abundance of hepatic antioxidant genes Mn-SOD (*p* = 0.048), PRDX3 (*p* = 0.002), and PRDX5 (*p* = 0.006) of piglets given a polydatin-supplemented diet. Polydatin alleviated the IUGR-induced reduction in the mRNA levels of SIRT3 (*p* = 0.003), Mn-SOD (*p* = 0.017), and PRDX3 (*p* < 0.001). Furthermore, no difference was found in the expression of SIRT1, NRF2, POLG, SSBP1, ACAA1, ACOX1, ACSL1, or TXNRD2 among the groups (*p* > 0.05).

## 4. Discussion

The liver performs a myriad of metabolic activities, which are tightly dependent on a large and constant energy supplied by mitochondria [33]. However, in IUGR animals, mitochondria dysfunction is frequently described as a typical pathological feature in different tissues and organs, including the liver [7,8]. The present work substantiated the findings of previous investigations [7,34,35,36], demonstrating that IUGR decreased ATP levels in the liver of piglets, along with the reduction in mitochondrial CS and α-KGDH activities. As the first and rate-limiting enzyme of the citrate cycle, CS controls the entry of acetyl-CoA into the cycle and is fundamental to mitochondrial oxidative metabolism and energy production [37]. Additionally, the decreases in mitochondrial complexes I and III activities were observed in the IUGR livers. Damage to one or more of the ETC complexes inevitably causes the shutdown of energy supply and thus undermines essential physiological functionalities of the liver, resulting in inferior resistance to various harmful stimuli, as well as a compromised growth performance [38].

Energy deficit may also be attributed to a decreased efficiency of mitochondrial biogenesis caused by IUGR. In this study, IUGR piglets showed lower mtDNA contents than their normal counterparts, probably because of the decreased SIRT1 activity and the low expression of nuclear genes that orchestrate mitochondrial biogenesis and fitness (i.e., PGC1α and TFAM). SIRT1 is a master factor that regulates mitochondrial biogenesis via PGC1α and is located both in the nucleus and cytoplasm [39]. The activated PGC1α can coactivate NRF1/2 and then promote the transcription of nuclear-encoded TFAM [40]. All of these factors control the amount and function of mtDNA and regulate the formation of mitochondrial components through a coordinated program [41]. Consistent with our findings, several reports have also shown that IUGR piglets exhibited a quantitative abnormality of mtDNA in the muscle [42], the small intestine [23], and the placenta [43]. Therefore, the defective mitochondrial biogenesis, combined with insufficient capacity of oxidative metabolism as mentioned above, may act in concert with the hepatic energy deficiency in the IUGR piglets.

Hepatic metabolism may have a fetal origin in the IUGR offspring [4]. Decreasing the metabolic efficiency of fetal liver may induce the limited nutrients being directed prevalently to essential organs, such as the brain and the heart [44]. This adaption is advantageous for the survival of the IUGR fetus in a hypoxic and nutrient deficient environment. However, these phenotype changes, if persistent into postnatal life when the availability of nutrients becomes sufficient, are detrimental to whole body energy homeostasis, and may well play a vital role in the evolution of insulin resistance and predisposition to metabolic sequelae later in life [45]. Actually, a growing body of epidemiological and experimental evidence reveals that fetal adaptations meant to counteract the malnutrition status in the short term would negatively affect long-term health [4,46,47]. Therefore, alleviating mitochondrial dysfunction with appropriate nutritional strategies is a promising approach to facilitating energy supply and maintaining the liver function and health status of IUGR individuals.

In this study, the IUGR piglets after polydatin treatment showed an increased ATP content in the liver compared to those fed a basal diet. A sufficient energy supply for the liver is not only of particular importance for supporting the increased metabolic demands postnatal, but also for controlling cell death and repairing tissue damage under adverse circumstances [38]. The mechanism by which polydatin improves hepatic energy supply may be due to its action to activate mitochondrial oxidative metabolism. First, polydatin significantly increased CS and α-KGDH activities and restored the IUGR-induced decrease in ICDH activity in the liver, signifying a greater efficiency of citrate cycle that generates reducing equivalents to drive ATP generation via oxidative phosphorylation. Correspondingly, complex I and ATP synthase activities were substantially increased after polydatin treatment, and the reduction in complex IV activity in the IUGR piglets was also reversed by polydatin supplementation. Similar results of increased mitochondrial CS and complexes I/II/III/IV/V activities have been observed in the myocardium from polydatin-treated mice [48]. These observations indicate that the benefits afforded by polydatin in the hepatic energy supply are probably mediated by the activation of the mitochondrial citrate cycle and subsequent oxidative phosphorylation.

Second, polydatin significantly increased the mRNA abundance of hepatic PPARα, and it concurrently upregulated CPT1A, FABP1, ACSL5, and HADHA transcript levels, all of which are the downstream targets of the PPARα signaling pathway. PPARα promotes fatty acid oxidation by transcriptionally activating a host of genes involved in lipid transport and lipolysis [49]. The metabolic products of fatty acid oxidation include acetyl-CoA and reducing equivalents, and these substrates can be further utilized by the mitochondrial citrate cycle or ETC complexes [50]. Therefore, the upregulation of PPARα and its downstream targets may be beneficial to increase fuel availability for the mitochondrial oxidative metabolism, which is probably another causative mechanism underlying the increased ATP production of the polydatin-treated livers.

Evidence is mounting that polydatin has the potential to activate SIRT3, both in vitro and in vivo [25,51]. In accord with these findings, we also found that polydatin attenuated IUGR-mediated decrease in the transcriptional expression of SIRT3. This effect may also favor the restoration of mitochondrial function in IUGR livers. SIRT3 is an important nuclear-encoded mitochondrial deacetylase, and it can activate enzymes related to glycolysis, fatty acid oxidation, the citrate cycle, and the ETC [52]. Thus, SIRT3 can act as a metabolic regulator of coupling substrate oxidation with the production of reducing equivalents to ATP generation, maximizing the efficiency of bioenergetic machinery [41]. Furthermore, SIRT3 regulates mitochondrial antioxidant defense, mitophagy, and apoptosis [52], and its activation appears to be a key event mediating the protective effects of polydatin against different harmful stimuli [25,51]. For example, in mouse models of cardiac dysfunction, polydatin could protect cardiomyocytes from myocardial infarction injury by improving mitochondrial biogenesis, enhancing autophagy, and inhibiting apoptosis, but these effects were largely abolished by SIRT3 knockout [48]. Similarly, in a recent research conducted by Wu et al. [51], the benefits of polydatin in sepsis also relied on SIRT3-mediated endothelial barrier protection.

So far, the mechanisms that polydatin activates SIRT3 remains largely incomplete. Nevertheless, several factors have been proposed to explain this association. The first involved the upregulation of PGC1α induced by polydatin treatment. PGC1α mediates ERRα binding to the SIRT3 promoter and thereby facilitates SIRT3 transcription [53]. Another possibility may be the action of polydatin to increase NADH oxidation. In this study, polydatin treatment shifted the hepatic NAD^+^/NADH couple to a more oxidized state, along with an increased content of NAD^+^, a co-substrate for multiple deacetylases, including SIRT1 and SIRT3 [41]. This effect may arise from the increased activity of hepatic mitochondrial complex I after polydatin treatment. Among the ETC components, complex I mediates the majority of oxygen consumption in mitochondria, being the major NADH oxidation site [54]. Activation of complex I can facilitate the transport of the electron flow to oxygen with an increase in NAD^+^ production [54]. However, additional research and in-depth investigations in this direction are still required to fully elucidate the mechanisms underlying the polydatin-mediated SIRT3 activation.

In addition to disrupting energy metabolism, mitochondrial dysfunction can also result in oxidative stress. As the primary source of intracellular ROS, the redox centers in mitochondrial ETC, and particularly in complexes I and III, cause the leakage of electrons from ETC, which then partially reduce molecule oxygen to superoxide anion, a precursor of most other ROS [55]. In the present study, the decreases in complexes I and III activities by IUGR may inhibit mitochondrial respiratory conductance. The ETC defects can exacerbate the leakage of electrons, intensify the ROS-emitting potential of mitochondria, and impair cellular redox-buffering capacity, ultimately perpetuating a cycle of oxygen radical-induced damage [56]. Of note, the ETC defects may create additional sites for increased electron pressure and leakage [41]. In the current study, a reduction in mitochondrial α-KGDH activity was noted for IUGR livers, which is also a potential producer of superoxide anion and hydrogen peroxide, particularly when NAD^+^ levels are diminished [8].

As expected, IUGR significantly increased hepatic apoptotic rates, probably due to the occurrence of mitochondrial dysfunction, as evidenced by the increased formation of mitochondrial superoxide anion and the decreased capacity of hepatic antioxidant defense. Mitochondrial ROS overproduction can directly stimulate the opening of mPTP, a channel across the mitochondrial inner and outer membranes, thereby allowing the free diffusion of matrix components (less than 1500 Da) into the cytosol [57]. Activation of the mPTP accentuates mitochondrial membrane potential collapse, disturbs mitochondrial functionality, and incurs substantial swelling of the organelle [58]. In this investigation, IUGR piglets had an obvious increase in hepatic mitochondrial swelling compared with their NBW counterparts. Mitochondrial swelling is one of the most important biomarkers for evaluating the probability for the mPTP to open [59]. The opening of mPTP can initiate apoptotic or necrotic cascades via the mitochondrial pathway, resulting in rapid cell death [60]. These results may therefore present another line of evidence for the increased activities of caspases 3 and 9 and the greater number of apoptotic cells in IUGR livers. Notably, the mPTP opening can also cause the loss of matrix NAD^+^/NADH ratio, and this decrease may cause a metabolic shift away from fatty acid oxidation towards lactate production, eventually stalling the citrate cycle and oxidative phosphorylation [61]. Taken together, mitochondrial functional decline, excessive ROS generation, and the activation of mitoptosis appear to be the major factors contributing to the liver injury of IUGR animals.

As a natural antioxidant, polydatin could assist in the recovery of mitochondrial lesion by scavenging free radicals and/or preventing their overproduction in different pathological conditions [25,62,63]. In the current study, the polydatin-treated IUGR piglets showed increases in Mn-SOD activity and expression levels of Mn-SOD, PRDX3, and PRDX5 in the liver, indicating an improvement in mitochondrial antioxidant function. Mn-SOD is present in the mitochondrial matrix and confers resistance to oxidative stress by mediating the reaction of the highly reactive, unstable superoxide anion to the less hazardous hydrogen peroxide, before it oxidizes cellular macromolecules [64]. PRDX3 and PRDX5 are also located in the mitochondria and catalyze the reduction of hydrogen peroxide and organic hydroperoxides, respectively, exerting a beneficial effect on mitochondrial protection against oxidative stress [65]. Similarly, Li et al. [62] found that polydatin restores severe shock-induced mitochondrial oxidative damage through promoting the protein expression of Mn-SOD. It has been demonstrated that polydatin serves as a potent activator of NRF2, a master redox-sensitive transcription factor that activates the expression of multiple downstream targets related to antioxidant and detoxification responses, including Mn-SOD [22]. Thus, the increased Mn-SOD activity may be due to the ability of polydatin to activate NRF2-mediated antioxidant signals.

In addition, the roles that polydatin played in increasing the efficiency of the mitochondrial respiratory chain may help decrease electron leakage to oxygen, and this effect could prevent the overproduction of superoxide radicals in the mitochondria [11]. In fact, a decrease in superoxide anion generation was noted in the mitochondria isolated from the livers of polydatin-treated IUGR piglets compared with those fed a basal diet. This effect, together with the increased activity of mitochondrial Mn-SOD and the upregulated expression of antioxidant genes, may exert synergistic actions in mitigating the hepatic mitochondrial lesions of IUGR piglets, with evidence of the decreases in mitochondrial swelling and caspase-9 activity, as well as an increase in mitochondrial membrane potential, in the liver of piglets fed a polydatin-supplemented diet. Moreover, polydatin alleviated hepatic oxidative injury prevented the excessive apoptosis of liver cells and inhibited plasma AST activity and T-Bil content, which may benefit from the ability of polydatin to restore mitochondrial redox balance.

Furthermore, NADPH oxidase (NOX) is also an important source of intracellular ROS. Cui et al. [66] have shown that NOX1 may be a key enzyme involved in the increased oxidative stress seen in the placenta with preeclampsia. Similarly, in a mouse model of IUGR, the protein expression of NOX4 is increased in the placenta [67,68]. These findings indicate that NOX may participate in the initiation and development of oxidative stress in IUGR individuals. Future work should determine whether NOX also affects redox status in the liver through this or related mechanisms and the potential action of polydatin to regulate NOX expression and/or its activity. This is of importance to broaden our understanding regarding the antioxidant property of polydatin.

## 5. Conclusions

The present study provides robust evidence that polydatin exerts beneficial roles in alleviating IUGR-induced liver injury and mitochondrial dysfunction in weanling piglets, which is ascribed to its potential to improve energy metabolism and redox balance. The findings of this study shed light on potential applications of polydatin as a mitochondrial protector to improve liver health in IUGR offspring.

## Figures and Tables

**Table 1:** Primer sequences of target and reference genes.

Gene	GenBank ID	Sequence (5′-3′)	Length (bp)
mt D-loop	AF_276923	GATCGTACATAGCACATATCATGTC	198
		GGTCCTGAAGTAAGAACCAGATG	
ACTB	DQ_452569	CCCCTCCTCTCTTGCCTCTC	74
		AAAAGTCCTAGGAAAATGGCAGAAG	
SIRT1	NM_001145750.2	AGTTGAAAGATGGCGGACGA	127
		CTCTCCGCGGTTTCTTGCG	
SIRT3	NM_001110057.1	TGGTGTCGTTCATCTGTTGCTG	117
		GGCACCGGGAGAAAAAGATATG	
PGC1α	NM_213963.2	TGTGCAACCAGGACTCTGTA	152
		CCACTTGAGTCCACCCAGAAA	
NRF1	XM_021078993.1	GAAGCTGTCCAGGGGCTTTA	116
		ATCCATGCTCTGCTACTGGG	
NRF2	NM_001185152.1	GGACAGCAGAAGTGATCCCC	97
		CAAAACCGTATCACTGGCCG	
ERRα	NM_001170521.1	GTCGCTACCCTCTGTGACCT	150
		GGCCACACCCAACACCAATA	
TFAM	NM_001130211.1	TGCTTTGTCTACGGGTGCAA	100
		ACTTCCACAAACCGCACAGA	
POLG	XM_001927064.4	CTGTCAGATGAGGGCGAGTG	133
		ACTTCTTCCGTCGTGACTTTCT	
SSBP1	XM_013985577.1	CTTTGAGGTAGTGCTGTGTCG	143
		CTCACCCCTGACGATGAAGAC	
PPARA	NM_001044526.1	GGCTGCTATCATTTGGTGCG	80
		GCACGATACCCTCCTGCATT	
CPT1A	NM_001129805.1	TGGTGTCCAAATACCTCGCC	145
		CCTCCGCTCGACACATACTC	
FABP1	NM_001004046.2	AGGGGACATCGGAAATCGTG	103
		TCACACTCCTCTCCCAAGGT	
ACAA1	XM_003132103.4	TTCAAGGACACCACCCCTGA	115
		GCTGAAGCACATTTCCCACG	
ACOX1	NM_001101028.1	GCTGTCACCATGAACCAGGA	166
		AGTTCAGGTCCTCATGCTGC	
ACSL1	NM_001167629.2	AGAAGGAAACCGAAGCCTCC	173
		AGAGTATCATCGGAGGAAGGACT	
ACSL5	NM_001195321.1	ACTTCAGAGCAGCTCACACC	123
		CCTTAGCTCTCCCCTCACCT	
HADHA	NM_213962.2	CGTCTACCAGGAGGGAGTGA	90
		ATGTCAGGCCGAGAGGGTAT	
Mn-SOD	NM_214127.2	GGCCTACGTGAACAACCTGA	126
		TGATTGATGTGGCCTCCACC	
PRDX3	NM_001244531.1	CATGTGAGTGCCGTTCCTTG	93
		AGACCACAGCACACTTGTCA	
PRDX5	NM_214144.1	GTGGTGGCATGTCTGAGTGT	170
		AGCCGTCGATTCCCAAAGAG	
TXNRD2	NM_001168702.1	TGCTACGACCTCCTGGTGA	110
		GGCGAAGGGCTCACATAGTC	
GAPDH	NM_001206359.1	CCAAGGAGTAAGAGCCCCTG	125
		AAGTCAGGAGATGCTCGGTG	

ACAA1, acetyl-CoA acyltransferase 1; ACTB, beta-actin; ACOX1, acyl-CoA oxidase 1; ACSL1, acyl-CoA synthetase long chain family member 1; ACSL5, acyl-CoA synthetase long-chain family member 5; CPT1α, carnitine palmitoyltransferase 1 alpha; ERRα, estrogen related receptor alpha; FABP1, fatty acid binding protein 1; GAPDH, glyceraldehyde-3-phosphate dehydrogenase; HADHA, hydroxyacyl-CoA dehydrogenase trifunctional multienzyme complex subunit alpha; Mn-SOD, manganese superoxide dismutase; NRF1, nuclear respiratory factor 1; NRF2, nuclear factor erythroid 2-related factor 2; PPARα, peroxisome proliferator activated receptor alpha; PGC1α, peroxisome proliferator activated receptor gamma coactivator 1 alpha; POLG, polymerase gamma; PRDX3, peroxiredoxin 3; PRDX5, peroxiredoxin 5; SIRT1, sirtuin 1; SIRT3, sirtuin 3; SSBP1, single-strand DNA-binding protein; TFAM, mitochondrial transcription factor A; TXNRD2, thioredoxin reductase 2.

**Table 2:** Effects of dietary polydatin supplementation on plasma biochemical parameters of normal birth weight and intra-uterine growth retarded weanling piglets.

Items	NC	IC	NP	IP	*p*-Value		
					BW	Diet	BW × Diet
GLU (mmol/L)	6.18 ± 1.31	4.57 ± 1.20	6.63 ± 1.40	6.70 ± 1.82	NS	0.041	NS
TG (mmol/L)	0.438 ± 0.384	0.350 ± 0.195	0.987 ± 0.466	0.678 ± 0.310	NS	0.006	NS
TC (mmol/L)	1.90 ± 1.09	1.62 ± 0.325	2.03 ± 0.766	2.07 ± 0.535	NS	NS	NS
TP (g/L)	46.7 ± 7.63	44.5 ± 6.09	52.0 ± 8.51	51.3 ± 6.80	NS	NS	NS
UN (mmol/L)	4.10 ± 1.19	6.30 ± 1.93	4.18 ± 2.52	5.58 ± 2.19	0.041	NS	NS
ALT (U/L)	40.8 ± 21.2	103 ± 74.2	30.3 ± 8.89	64.7 ± 39.8	0.014	NS	NS
AST (U/L)	85.5 ± 43.9	172 ± 72.4	44.2 ± 22.8	61.2 ± 38.2	0.015	0.001	NS
T-Bil (μmol/L)	3.88 ± 2.74	9.03 ± 6.10	1.98 ± 3.51	3.50 ± 3.29	NS	0.039	NS

ALT, alanine aminotransferase; AST, aspartate aminotransferase; BW, birth weight; GLU, glucose; IC, intra-uterine growth retarded piglets fed a basal diet; IP, intra-uterine growth retarded piglets fed a polydatin-supplemented diet; NC, normal birth weight piglets fed a basal diet; NP, normal birth weight piglets fed a polydatin-supplemented diet; NS, non-significant; T-Bil, total bilirubin; TC, total cholesterol; TG, triglycerides; TP, total protein; UN, urea nitrogen. Results are expressed as mean values and standard deviations (*n* = 6).

**Table 3:** Effects of dietary polydatin supplementation on apoptotic rate and caspase activities in the liver of normal birth weight and intra-uterine growth retarded weanling piglets.

Items	NC	IC	NP	IP	*p*-Value		
					BW	Diet	BW × Diet
Apoptosis (%)	1.18 ± 0.410 ^b^	3.86 ± 1.31 ^a^	1.06 ± 0.570 ^b^	1.89 ± 0.785 ^b^	<0.001	0.006	0.014
Caspase-3 (U/mg protein)	23.9 ± 7.94	40.3 ± 8.57	26.7 ± 10.1	29.0 ± 7.78	0.016	NS	NS
Caspase-8 (U/mg protein)	5.41 ± 1.92	6.16 ± 1.68	4.78 ± 1.69	4.96 ± 2.00	NS	NS	NS
Caspase-9 (U/mg protein)	13.8 ± 1.73	18.0 ± 2.88	10.3 ± 4.41	13.1 ± 3.10	0.013	0.004	NS

BW, birth weight; IC, intra-uterine growth retarded piglets fed a basal diet; IP, intra-uterine growth retarded piglets fed a polydatin-supplemented diet; NC, normal birth weight piglets fed a basal diet; NP, normal birth weight piglets fed a polydatin-supplemented diet; NS, non-significant. ^a,b^ Data with different superscript letters were significantly different (*p* < 0.05). Results are expressed as mean values and standard deviations (*n* = 6).

**Table 4:** Effects of dietary polydatin supplementation on oxidative metabolic enzyme activities and energy metabolite contents in the liver of normal birth weight and intra-uterine growth retarded weanling piglets.

Items	NC	IC	NP	IP	*p*-Value		
					BW	Diet	BW × Diet
Citrate cycle enzyme activities							
CS (U/mg protein)	20.9 ± 12.3	10.3 ± 3.48	27.5 ± 9.16	19.9 ± 5.22	0.014	0.026	NS
ICDH (U/mg protein)	8.13 ± 2.22 ^a,b^	5.56 ± 2.76 ^b^	7.52 ± 1.38 ^a,b^	9.65 ± 1.87 ^a^	NS	NS	0.013
α-KGDH (U/mg protein)	2.27 ± 0.802	1.38 ± 0.665	3.26 ± 1.38	2.22 ± 1.17	0.035	0.046	NS
MDH (U/mg protein)	5.28 ± 1.45	5.76 ± 1.84	5.50 ± 1.31	5.14 ± 3.52	NS	NS	NS
Respiratory chain complex activities							
Complex I (U/mg protein)	15.7 ± 9.32	7.88 ± 3.97	23.2 ± 9.34	16.1 ± 5.50	0.023	0.017	NS
Complex II (U/mg protein)	8.11 ± 4.52	6.61 ± 5.25	10.5 ± 4.08	11.8 ± 6.40	NS	NS	NS
Complex III (U/mg protein)	7.39 ± 3.33	4.91 ± 1.65	8.66 ± 2.39	6.54 ± 2.58	0.039	NS	NS
Complex IV (U/mg protein)	31.0 ± 12.5 ^a,b^	18.6 ± 7.33 ^b^	25.1 ± 6.11 ^a,b^	32.6 ± 5.40 ^a^	NS	NS	0.008
ATP synthase (U/mg protein)	39.4 ± 10.6	29.3 ± 8.74	50.5 ± 12.6	43.7 ± 18.4	NS	0.027	NS
Energy metabolite contents							
ATP (μmol/g wet weight)	25.6 ± 10.1	15.6 ± 6.96	30.3 ± 2.29	23.7 ± 7.77	0.012	0.045	NS
NAD^+^ (μmol/g wet weight)	1.33 ± 0.382	1.07 ± 0.184	1.66 ± 0.228	1.47 ± 0.309	NS	0.005	NS
NADH (μmol/g wet weight)	0.559 ± 0.363	0.614 ± 0.138	0.598 ± 0.142	0.550 ± 0.0895	NS	NS	NS
NAD ^+^ /NADH (μmol/μmol)	2.73 ± 0.829	1.77 ± 0.283	2.90 ± 0.686	2.73 ± 0.664	0.045	0.047	NS

α-KGDH, alpha ketoglutarate dehydrogenase; ATP, adenosine triphosphate; BW, birth weight; CS, citrate synthase; IC, intra-uterine growth retarded piglets fed a basal diet; ICDH, isocitrate dehydrogenase; IP, intra-uterine growth retarded piglets fed a polydatin-supplemented diet; NAD^+^, nicotinamide adenine dinucleotide; NADH, reduced form of nicotinamide adenine dinucleotide; NC, normal birth weight piglets fed a basal diet; NP, normal birth weight piglets fed a polydatin-supplemented diet; NS, non-significant; MDH, malic dehydrogenase. ^a,b^ Data with different superscript letters were significantly different (*p* < 0.05). Results are expressed as mean values and standard deviations (*n* = 6).

**Table 5:** Effects of dietary polydatin supplementation on antioxidant enzyme activities and redox metabolite contents in the liver of normal birth weight and intra-uterine growth retarded weanling piglets.

Items	NC	IC	NP	IP	*p*-Value		
					BW	Diet	BW × Diet
Antioxidant enzyme activities							
Cu/Zn-SOD (U/mg protein)	66.5 ± 15.0	57.5 ± 15.5	87.3 ± 23.1	61.4 ± 9.89	0.018	NS	NS
Mn-SOD (U/mg protein)	36.0 ± 12.5 ^a,b^	19.6 ± 8.16 ^b^	37.1 ± 16.8 ^a,b^	48.6 ± 21.9 ^a^	NS	0.029	0.041
GPx (U/mg protein)	95.4 ± 44.4	85.2 ± 28.8	126 ± 42.3	88.1 ± 19.0	NS	NS	NS
GR (U/g protein)	9.54 ± 3.08	8.54 ± 2.66	11.3 ± 4.22	8.22 ± 2.82	NS	NS	NS
CAT (U/g protein)	115 ± 38.4	116 ± 44.0	106 ± 39.8	134 ± 47.5	NS	NS	NS
Redox metabolite contents							
GSH (μmol/g protein)	5.94 ± 2.18	2.77 ± 1.09	5.80 ± 3.76	3.91 ± 2.83	0.030	NS	NS
8-OHdG (ng/mg DNA)	1.69 ± 0.738 ^b^	3.04 ± 0.380 ^a^	1.77 ± 0.399 ^b^	1.76 ± 0.481 ^b^	0.005	0.011	0.004
PC (nmol/mg protein)	1.51 ± 0.443	2.39 ± 0.971	1.56 ± 0.306	1.61 ± 0.431	NS	NS	NS
MDA (nmol/mg protein)	2.56 ± 0.772 ^b^	5.28 ± 1.99 ^a^	2.89 ± 1.53 ^b^	2.70 ± 1.24 ^b^	0.046	NS	0.024

BW, birth weight; CAT, catalase; Cu/Zn-SOD, copper/zinc superoxide dismutase; GPx, glutathione peroxidase; GR, glutathione reductase; GSH, reduced glutathione; IC, intra-uterine growth retarded piglets fed a basal diet; IP, intra-uterine growth retarded piglets fed a polydatin-supplemented diet; NC, normal birth weight piglets fed a basal diet; NP, normal birth weight piglets fed a polydatin-supplemented diet; NS, non-significant; MDA, malondialdehyde; Mn-SOD, manganese superoxide dismutase; 8-OHdG, 8-hydroxy-2-deoxyguanosine; PC, protein carbonyl. ^a,b^ Data with different superscript letters were significantly different (*p* < 0.05). Results are expressed as mean values and standard deviations (*n* = 6).

**Table 6:** Effects of dietary polydatin supplementation on the expression of genes related to energy metabolism and mitochondrial function in the liver of normal birth weight and intrauterine growth retarded weanling piglets.

Items	NC	IC	NP	IP	*p*-Value		
					BW	Diet	BW × Diet
SIRT1	1.00 ± 0.758	0.877 ± 0.668	1.04 ± 0.891	1.26 ± 0.692	NS	NS	NS
SIRT3	1.00 ± 0.366 ^a^	0.380 ± 0.0922 ^b^	0.819 ± 0.257 ^a,b^	1.14 ± 0.487 ^a^	NS	0.048	0.003
PGC1α	1.00 ± 0.358	0.567 ± 0.227	1.89 ± 0.898	1.23 ± 0.482	0.025	0.003	NS
NRF1	1.00 ± 0.252	1.27 ± 0.454	2.39 ± 1.64	1.83 ± 0.978	NS	0.025	NS
NRF2	1.00 ± 0.678	1.10 ± 0.394	1.24 ± 0.749	1.44 ± 0.777	NS	NS	NS
ERRα	1.00 ± 0.283	0.694 ± 0.324	1.29 ± 0.381	1.36 ± 0.594	NS	0.010	NS
TFAM	1.00 ± 0.424	0.538 ± 0.308	1.23 ± 0.248	0.871 ± 0.257	0.005	0.043	NS
POLG	1.00 ± 0.820	1.10 ± 1.06	1.49 ± 1.27	0.823 ± 0.344	NS	NS	NS
SSBP1	1.00 ± 0.202	1.35 ± 0.817	0.939 ± 0.452	0.896 ± 0.267	NS	NS	NS
PPARα	1.00 ± 0.392	0.453 ± 0.106	2.59 ± 1.13	2.95 ± 2.05	NS	<0.001	NS
CPT1α	1.00 ± 0.301	0.571 ± 0.252	2.03 ± 1.54	2.46 ± 1.10	NS	0.001	NS
FABP1	1.00 ± 0.104	0.604 ± 0.178	1.69 ± 0.563	1.45 ± 0.273	0.028	<0.001	NS
ACAA1	1.00 ± 0.165	0.882 ± 0.333	0.951 ± 0.216	0.824 ± 0.180	NS	NS	NS
ACOX1	1.00 ± 0.330	0.828 ± 0.396	0.785 ± 0.295	1.10 ± 0.409	NS	NS	NS
ACSL1	1.00 ± 0.205	0.629 ± 0.214	1.07 ± 0.674	1.08 ± 0.168	NS	NS	NS
ACSL5	1.00 ± 0.348	1.04 ± 0.529	1.70 ± 0.729	2.14 ± 0.640	NS	0.001	NS
HADHA	1.00 ± 0.306	0.811 ± 0.293	1.81 ± 0.621	1.03 ± 0.702	0.031	0.023	NS
Mn-SOD	1.00 ± 0.261 ^a,b^	0.560 ± 0.186 ^b^	0.943 ± 0.350 ^a,b^	1.10 ± 0.307 ^a^	NS	0.048	0.017
PRDX3	1.00 ± 0.267 ^a,b^	0.414 ± 0.130 ^c^	0.815 ± 0.271 ^b,c^	1.36 ± 0.333 ^a^	NS	0.002	<0.001
PRDX5	1.00 ± 0.247	0.704 ± 0.222	1.46 ± 0.560	1.10 ± 0.194	0.028	0.006	NS
TXNRD2	1.00 ± 0.296	0.806 ± 0.608	1.49 ± 0.635	0.881 ± 0.498	NS	NS	NS

ACAA1, acetyl-CoA acyltransferase 1; ACTB, beta-actin; ACOX1, acyl-CoA oxidase 1; ACSL1, acyl-CoA synthetase long chain family member 1; ACSL5, acyl-CoA synthetase long-chain family member 5; BW, birth weight; CPT1α, carnitine palmitoyltransferase 1 alpha; ERRα, estrogen related receptor alpha; FABP1, fatty acid binding protein 1; GAPDH, glyceraldehyde-3-phosphate dehydrogenase; HADHA, hydroxyacyl-CoA dehydrogenase trifunctional multienzyme complex subunit alpha; IC, intra-uterine growth retarded piglets fed a basal diet; IP, intra-uterine growth retarded piglets fed a polydatin-supplemented diet; Mn-SOD, manganese superoxide dismutase; NC, normal birth weight piglets fed a basal diet; NP, normal birth weight piglets fed a polydatin-supplemented diet; NRF1, nuclear respiratory factor 1; NRF2, nuclear factor erythroid 2-related factor 2; NS, non-significant; PPARα, peroxisome proliferator activated receptor alpha; PGC1α, peroxisome proliferator activated receptor gamma coactivator 1 alpha; POLG, polymerase gamma; PRDX3, peroxiredoxin 3; PRDX5, peroxiredoxin 5; SIRT1, sirtuin 1; SIRT3, sirtuin 3; SSBP1, single-strand DNA-binding protein; TFAM, mitochondrial transcription factor A; TXNRD2, thioredoxin reductase 2. ^a,b,c^ Data with different superscript letters were significantly different (*p* < 0.05). Results are expressed as mean values and standard deviations (*n* = 6).

## Data Availability

Data are available within the article and supplementary materials.

## Supplementary Materials

The following supporting information can be downloaded at: https://www.mdpi.com/article/10.3390/antiox11040666/s1, Table S1: Composition and nutrient levels of the diet; Table S2: Effects of resveratrol and polydatin on superoxide dismutase activities of AML-12 cells under the conditions of oxidative stress; Figure S1: Certificate of analysis of the polydatin; Figure S2: 2,2-Diphenyl-1-picrylhydrazyl radical scavenging effects of butylated hydroxyanisole, butylated hydroxytoluene, vitamin C, vitamin E, resveratrol, and polydatin at 10–400 μM; Figure S3: Superoxide radical scavenging activity of polydatin at 25–1000 μM; Figure S4: Effects of resveratrol, polydatin, and piceatannol on the viability of AML-12 cells under the conditions of oxidative stress.
