# Supplementary material for: Polydatin Attenuates Intra-Uterine Growth Retardation-Induced Liver Injury and Mitochondrial Dysfunction in Weanling Piglets by Improving Energy Metabolism and Redox Balance
Source: Antioxidants (Basel). 2022 Mar 30;11(4):666. doi: 10.3390/antiox11040666 (PMC9028342; doi:10.3390/antiox11040666)
Supplement: Supplementary file 1 [file antioxidants-11-00666-s001.zip › antioxidants-1611260-supplementary.pdf]

Supporting Information

1. Materials and Methods

1.1. Chemicals

Polydatin used in the present study was purchased from Aladdin Reagents Co., Ltd. (Shanghai, China), and it was isolated from the Chinese herb *Polygonum cuspidatum*. According to the certificate of analysis, the purity of polydatin was 97.36%, which was determined by high-performance liquid chromatography (Figure S1).

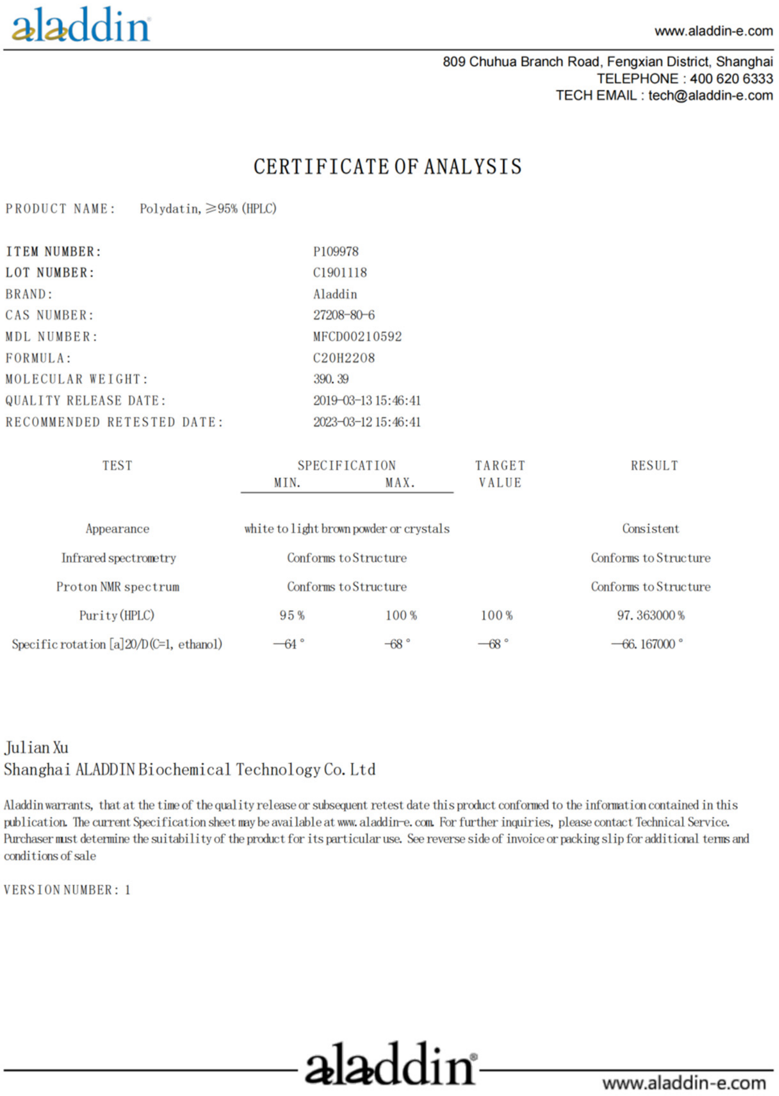

aladdin

www.aladdin-e.com

809 Chuhua Branch Road, Fengxian District, Shanghai
TELEPHONE : 400 620 6333
TECH EMAIL : tech@aladdin-e.com

CERTIFICATE OF ANALYSIS

PRODUCT NAME: Polydatin, ≥95% (HPLC)

ITEM NUMBER: P109978
LOT NUMBER: C1901118
BRAND: Aladdin
CAS NUMBER: 27208-80-6
MDL NUMBER: MFCD00210592
FORMULA: C20H22O8
MOLECULAR WEIGHT: 390.39
QUALITY RELEASE DATE: 2019-03-13 15:46:41
RECOMMENDED RETESTED DATE: 2023-03-12 15:46:41

TEST	SPECIFICATION MIN.	SPECIFICATION MAX.	TARGET VALUE	RESULT
Appearance	white to light brown powder or crystals			Consistent
Infrared spectrometry	Conforms to Structure			Conforms to Structure
Proton NMR spectrum	Conforms to Structure			Conforms to Structure
Purity (HPLC)	95 %	100 %	100 %	97.363000 %
Specific rotation [a]20/D (C=1, ethanol)	−64 °	−68 °	−68 °	−66.167000 °

Julian Xu
Shanghai ALADDIN Biochemical Technology Co. Ltd

Aladdin warrants, that at the time of the quality release or subsequent retest date this product conformed to the information contained in this publication. The current Specification sheet may be available at www.aladdin-e.com. For further inquiries, please contact Technical Service. Purchaser must determine the suitability of the product for its particular use. See reverse side of invoice or packing slip for additional terms and conditions of sale

VERSION NUMBER: 1

aladdin
www.aladdin-e.com

Figure S1. Certificate of analysis of the polydatin.

Resveratrol was purchased from BOC Sciences (Shirley, NY, USA; purity ≥ 99%, HPLC). 2,2-Dipheny-l-picrylhydrazyl (DPPH), nitro blue tetrazolium (NBT), phenazine methosulfate (PMS), nicotinamide adenine dinucleotide (NADH), butylated hydroxyanisole, and butylated hydroxytoluene were purchased from Sigma Chemical Co., Ltd. (Shanghai, China). All other chemicals used in the present study were obtained from Shanghai Chemical Agents Co., Ltd. (Shanghai, China) and were of analytical grade.

1.2. Composition and Nutrient Levels of the Basal Diet

The basal diet was formulated based on the recommendations of the National Research Council (2012) for piglets weighing 5–10 kg (Table S1)

Table S1. Composition and nutrient levels of the basal diet.

Items	Contents
Ingredient (%)	
Maize	62.78
Soybean meal	15.00
Fermented soybean meal	7.00
Extruded soybean	7.00
Soy protein isolate	1.30
Soyabean oil	2.00
$CaHPO_4$	1.80
Limestone	0.80
Salt	0.35
L-lysine-HCl (78.0%)	0.52
L-methionine	0.13
L-threonine	0.15
L-isoleucine	0.10
L-tryptophan	0.01
L-histidine	0.01
Calcium propionate (50.0%)	0.05
Premix[1]	1.00
Total	100.00
Nutrient levels[2]	
Digestible energy (Mcal/kg)	3.47
Crude protein (%)	20.36
Total lysine (%)	1.51
Total methionine (%)	0.46
Total methionine + cystine (%)	0.86
Total threonine (%)	0.94
Total tryptophan (%)	0.40
Total histidine (%)	0.77
Total isoleucine (%)	0.79
Total valine (%)	1.20
Total calcium (%)	0.82
Total phosphorus (%)	0.65

CP, crude protein.
[1]Provide the following per kg complete diet: Vitamin A, 8,000 IU; Vitamin D_3, 3,000 IU; Vitamin E, 20 IU; Vitamin K_3, 3 mg; Vitamin B_1, 2 mg; Vitamin B_2, 5 mg; Vitamin B_6, 7 mg; Vitamin B_{12}, 0.02 mg; Niacin, 30 mg; Pantothenic acid, 15 mg; Folic acid, 0.3 mg; Biotin, 0.08 mg; Choline chloride, 500 mg; Fe (from ferrous sulfate), 110 mg; Cu (from copper sulfate), 7 mg; Mn (from manganese sulfate), 5 mg; Zn (from zinc sulfate), 110 mg; I (from calcium iodate), 0.3 mg; Se (from sodium selenite), 0.3 mg.
[2]All nutrient levels were analyzed values, except digestible energy.

1.3. DPPH Radical Scavenging Activity

DPPH radical scavenging activity was measured according to the method of Liu et al. [32]. Briefly, DPPH was dissolved in ethanol to a 0.1 mM solution. Aliquots of DPPH solution and sample solutions at various concentrations (10–400 μM) were mixed and shaken vigorously. Then the absorbance was determined at 517 nm after incubation for 30 min in the dark at room temperature. The percent DPPH scavenging effect was calculated using the following equation:

$$\text{DPPH scavenging effect (\%)} = \frac{A_{\text{control}} - A_{\text{sample}}}{A_{\text{control}}} \times 100\%$$

$A_{control}$ was the absorbance of the control reaction and A_{sample} was the absorbance in the presence of sample or reference substances.

1.4. Superoxide radical scavenging activity

Superoxide anion ($O_2^{\bullet-}$) scavenging activity was determined according to the method of Chen and Yen [68]. $O_2^{\bullet-}$ was generated in a non-enzymic system and determined by a spectrophotometric measurement for reduction of NBT. Briefly, PMS, NADH, and NBT were dissolved in phosphate buffer (0.1 M, pH = 7.4) to a 60, 468, and 150 μM solutions, respectively. The reaction mixture contained 1 mL of polydatin solution at various concentrations (25–1000 μM), 1 mL of PMS solution, 1 mL of NADH solution, and 1 mL of NBT solution, and were incubated at ambient temperature for 5 min. After that, the color was read at 560 nm against blank samples. The percentage inhibition of $O_2^{\bullet-}$ generation was calculated using the following formula:

$$O_2^{\bullet-} \text{ scavenging effect (\%)} = \frac{A_{\text{control}} - A_{\text{sample}}}{A_{\text{control}}} \times 100\%$$

$A_{control}$ was the absorbance of the control reaction and A_{sample} was the absorbance in the presence of sample or reference substances.

1.5. Cell Culture and Treatment

The alpha mouse liver 12 (AML-12) cell line was obtained from American Type Culture Collection (Manassas, VA, USA) and cultured in DMEM/F-12 medium (Gibco-BRL, Grand Island, NY, USA) supplemented with 10% fetal bovine serum (Gibco-BRL), 100 U/mL penicillin and streptomycin (Gibco-BRL), Insulin-transferrin-selenium Liquid Media Supplement (Sigma-Aldrich), and 40 ng/mL dexamethasone at 37°C in a humidified O_2/CO_2 (19:1) atmosphere. For the experiments, AML12 cells were pre-treated with resveratrol, polydatin, and piceatannol at different concentrations (0, 0.5, 1.0, 2.5, 5.0, and 10.0 μM) for 24 h and then exposed to 0.5 mM hydrogen peroxide (H_2O_2) for another 1 h.

1.6. Cell Viability Assay

The viability of the AML-12 cells was tested by planting the cells in 96-well microplates at a density of 1×10^4 cells per well and subjecting them to various treatments. Cell viability was then determined by incubating the cells with Cell Counting Kit-8 solution (10 μL/well; Yeasen, Shanghai, China) for 1.5 h at 37°C, and the absorbance at 450 nm was measured by a Multiskan SkyHigh (Thermo Fisher Scientific, Waltham, MA, USA).

1.7. Determination of Copper/Zinc Superoxide Dismutase (Cu/Zn-SOD) and Manganese Superoxide Dismutase (Mn-SOD) Activities

The AML-12 cells were pre-treated with resveratrol and polydatin at a dose of 5.0 μM for 24 h and then exposed to 0.5 mM H_2O_2 for another 1 h. After that, AML12 cells were homogenized with phosphate buffer saline and centrifuged at 3000 × *g* at 4°C for 10 min. Subsequently, the supernatants were collected for determination of the activities of Cu/Zn-SOD and Mn-SOD. The detection kits were supplied by Nanjing Jiancheng Bioengineering Institute (Nanjing, Jiangsu, China).

1.8. Statistical Analysis

All statistical analyses were conducted using SPSS Statistics (Version 26.0, IBM, Armonk, NY, USA). One-way ANOVA and Tukey's post hoc tests for pair comparisons were conducted for the data obtained from cellular experiments. Difference was considered significant at $P<0.05$. Results were expressed as mean values and standard deviations.

2. Results

2.1. DPPH radical scavenging activity

DPPH is a stable nitrogen radical that has been widely used to evaluate radical quenching capacities of natural antioxidants and some other plant extracts. This study compared the *in vitro* antioxidant capacities of resveratrol and polydatin. Meanwhile, the water-soluble vitamin C, the fat-soluble vitamin E, and two synthetic antioxidants (i. e., butylated hydroxyanisole and butylated hydroxytoluene) were adopted as reference radical scavengers.

Resveratrol and polydatin were found to scavenge DPPH radicals with a dose-dependent manner *in vitro* (Figure S2). At the concentration of 400 μM, both resveratrol and polydatin exhibited higher DPPH radical scavenging activities than vitamin C and butylated hydroxytoluene, but their scavenging abilities were still lower than vitamin E and butylated hydroxyanisole.

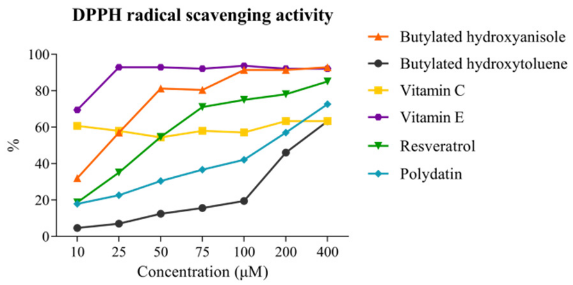

Figure S2. 2,2-Diphenyl-1-picrylhydrazyl radical scavenging effects of butylated hydroxyanisole, butylated hydroxytoluene, vitamin C, vitamin E, resveratrol, and polydatin at 10–400 μM. DPPH, 2,2-diphenyl-1-picrylhydrazyl.

2.1. $O_2^{\bullet-}$ scavenging activity

$O_2^{\bullet-}$ is one of the most representative physiological radicals and it is mainly derived from the non-enzymatic electron transfers in mitochondria and the enzymatic system mediated by NADPH oxidases. The effect of $O_2^{\bullet-}$ can be magnified since it is a precursor of other kinds of free radicals and oxidizing agents. Thus, a PMS–NBT assay was carried out to evaluate the ability of polydatin to quench $O_2^{\bullet-}$.

In this study, the $O_2^{\bullet-}$ scavenging activity of polydatin was raised linearly as the concentration increased (Figure S3). At the concentration of 1000 μM, the $O_2^{\bullet-}$ scavenging activity of polydatin was 85.6 ± 0.8%. Thus, these results indicate that polydatin may serve as a scavenger of $O_2^{\bullet-}$.

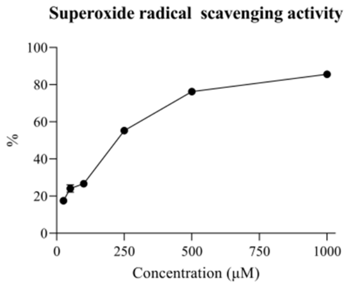

Figure S3. Superoxide radical scavenging activity of polydatin at 25–1000 μM.

2.2. Effects of resveratrol, polydatin, and piceatannol on the viability of AML-12 cells upon oxidative stress

To assess the cytoprotective effects of resveratrol, polydatin, and piceatannol, AML-12 cells were treated with different concentrations (0, 0.5, 1.0, 2.5, 5.0, and 10.0 μM) of these compounds for 24 h, followed by 0.5 mM H_2O_2 for another 1 h. The results indicated that, compared with the H_2O_2-only group, resveratrol (5.0 and 10.0 μM), polydatin (1.0, 2.5, 5.0, and 10.0 μM), and piceatannol (10.0 μM) significantly increased the viability of AML-12 cells ($P<0.05$; Figure S4). Based on these observations, 5.0 μM resveratrol and polydatin were selected for subsequent determination of superoxide dismutase activities.

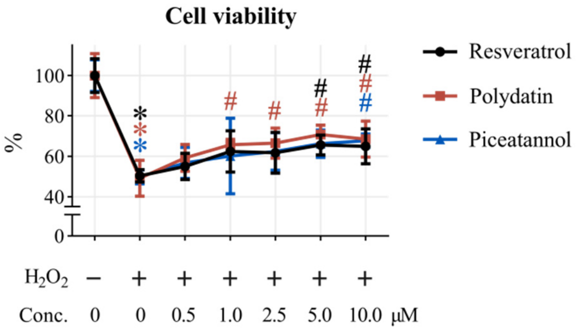

Figure S4. Effects of resveratrol, polydatin, and piceatannol on the viability of AML-12 cells under the conditions of oxidative stress. Significant difference is depicted as $^{*}P<0.05$ compared with control group, $^{\#}P<0.05$ compared with H_2O_2 group. Results are expressed as mean values and standard deviations (n = 6).

2.3. Effects of resveratrol and polydatin on superoxide dismutase activities of AML-12 cells upon of oxidative stress

Subsequently, the activities of Cu/Zn-SOD and Mn-SOD were determined to evaluate the antioxidant effects of resveratrol and polydatin. The data showed that H_2O_2 stimulation tended to inhibit the activity of Mn-SOD ($P=0.068$; Table S2) in the AML-12 cells. By contrast, a tendency towards increased Mn-SOD activity ($P=0.090$) was observed in the H_2O_2-exposed cells pre-treated with 5.0 μM polydatin. However, this effect was absent in the resveratrol-treated group ($P>0.10$). These findings might be explained by the difference in cellular uptake efficiency between these stilbenes. Polydatin is more efficiently absorbed than resveratrol, since it can enter cells via active mechanism using glucose carriers [19]. This property may facilitate the action of polydatin to activate the antioxidant response of AML-12 cells.

Table S2. Effects of resveratrol and polydatin on superoxide dismutase activities of AML-12 cells under the conditions of oxidative stress.

Items	CON (Group I)	H_2O_2 (Group II)	H_2O_2-RSV (Group III)	H_2O_2-PD (Group IV)	*P*-value		
					I *vs.* II	II *vs.* III	II *vs.* IV
Cu/Zn-SOD (U/mg protein)	219±26.0	182±24.0	202±22.9	204±13.8	NS	NS	NS
Mn-SOD (U/mg protein)	61.5±3.68	48.3±2.51	55.6±3.34	60.6±2.88	NS	NS	NS

CON, the AML-12 cells without hydrogen peroxide stimulation or stilbene treatment; Cu/Zn-SOD, copper/zinc superoxide dismutase; H_2O_2, the hydrogen peroxide-exposed AML-12 cells without stilbene treatment; H_2O_2-RSV, the hydrogen peroxide-exposed AML-12 cells treated with 5.0 μM resveratrol; H_2O_2-PD, the hydrogen peroxide-exposed AML-12 cells treated with 5.0 μM polydatin; Mn-SOD, manganese superoxide dismutase. Results are expressed as mean values and standard deviations (n = 3).
